# Green Approach: ‘‘A Forwarding Step for Curing Leishmaniasis—A Neglected Tropical Disease’’

**DOI:** 10.3389/fmolb.2021.655584

**Published:** 2021-05-28

**Authors:** Lakshika Sharma, Mamta Dhiman, Abhijeet Singh, M. M. Sharma

**Affiliations:** Department of Biosciences, Manipal University Jaipur, Jaipur, India

**Keywords:** leishmaniasis, natural sources, nanobiotech, green route, nanoformulations

## Abstract

The present review focuses on a dreaded vector-mediated leishmaniasis, with the existing therapeutic approaches including a variety of drugs along with their limitations, the treatment with natural compounds, and different types of metal/metal oxide nanoparticles (NPs). As evidenced, various metallic NPs, comprising silver, silver oxide, gold, zinc oxide, titanium, lead oxide, *etc.*, played a curative role to treat leishmaniasis, are also presented. Keeping in view the advance success of vaccines against the prevalent dreaded diseases in the past and the present scenario, efforts are also being made to develop vaccines based on these NP formulations.

## Introduction

Neglected tropical diseases (NTDs) are contagious diseases that cause substantial illness in more than one billion people globally (Maheshwari and Bandyopadhyay, 2020). Various parasite-mediated diseases comprising giardiasis, Chagas disease, Babesiosis, toxoplasmosis, leishmaniasis, *etc.*, befall in animals and further spread to human population (Oryan, 2015; Hotez et al., 2020). Leishmaniasis is one of the NTDs considered as imperative parasitic diseases, commonly caused by an etiologic agent *Leishmania*, a genus of trypanosomes. Leishmaniasis is located in the ninth place of the global burden of disease among individual infectious diseases. More than 22 species of infectious *Leishmania* have been reported (Maheshwari and Bandyopadhyay, 2020). *Leishmania* are transmitted to mammals through the bite of infected female sandflies belonging to *Lutzomyia* and *Phlebotomus* (Oryan and Akbari, 2016). It is endemic in 98 nations of the world, where more than 350 million people are at risk and more than 12 million cases of infection have been reported (Verma and Dey, 2004; Mcgwire and Satoskar, 2014). Based on the species and intensity of infection to the host, it has been classified into cutaneous leishmaniasis (CL), mucocutaneous leishmaniasis (MCL), and visceral leishmaniasis (VL) forms. Considering these classes, CL caused *by L. aethiopica* is most commonly found in human population and reported to infect 6,00,000 to one million people annually all around the world. It causes severe symptoms like ulcers, serious disabilities, and life-long marks (Surur et al., 2020). Previously, various chemical drugs including liposomal amphotericin B, amphotericin B, pentamidine, pentavalent antimonials, miltefosine, and paromomycin have been practiced against *Leishmania*. Among these drugs, pentavalent antimonials (sodium stibogluconate and meglumine) are existing chemical drugs, and they are a major therapeutic source to treat *Leishmania* infection. However, current treatment practices are associated with certain side effects like high toxicity, high cost, and most importantly, development of drug resistance. Hence, there is an instantaneous necessity to innovate new, harmless, and efficient prevention therapies to overcome these limitations (Mitropoulos et al., 2010). Currently, various approaches are involved to control the elevated level of infection, including nanoformulations and targeted drug delivery using nanocarriers as well as with the aid of particular bioactive compound obtained from plants (Javed et al., 2020; Santana et al., 2020). Inventions in nanoscience greatly contribute to overcome the problems allied with the treatment of infectious diseases. Owing to the small size of NPs (1–100 nm), the ability to penetrate easily into the cells, extensive circulation within the body, and the efficient targeted drug delivery system, they can be reflected as a better medication to treat endemic leishmaniasis (Ebrahimi et al., 2017). Further, plant-based nanoparticles have been reported as a successful approach for the preclusion of microbial infections as well as in the treatment of leishmaniasis (Alti et al., 2020). Owing to the green and eco-friendly nature, cost effectiveness, less hazardous nature, and involvement of phytoconstituents for capping and stabilization, plant-based metal oxide nanoparticles (NPs) (silver, zinc, nickel, iron, *etc.*) have been in use to cure leishmanial infection (Ismail et al., 2019; Alti et al., 2020). The present review focuses on the use of plant-based natural products, the phytosynthesized NPs, to cure *Leishmania*.

## Life Cycle

**FIGURE 1:**
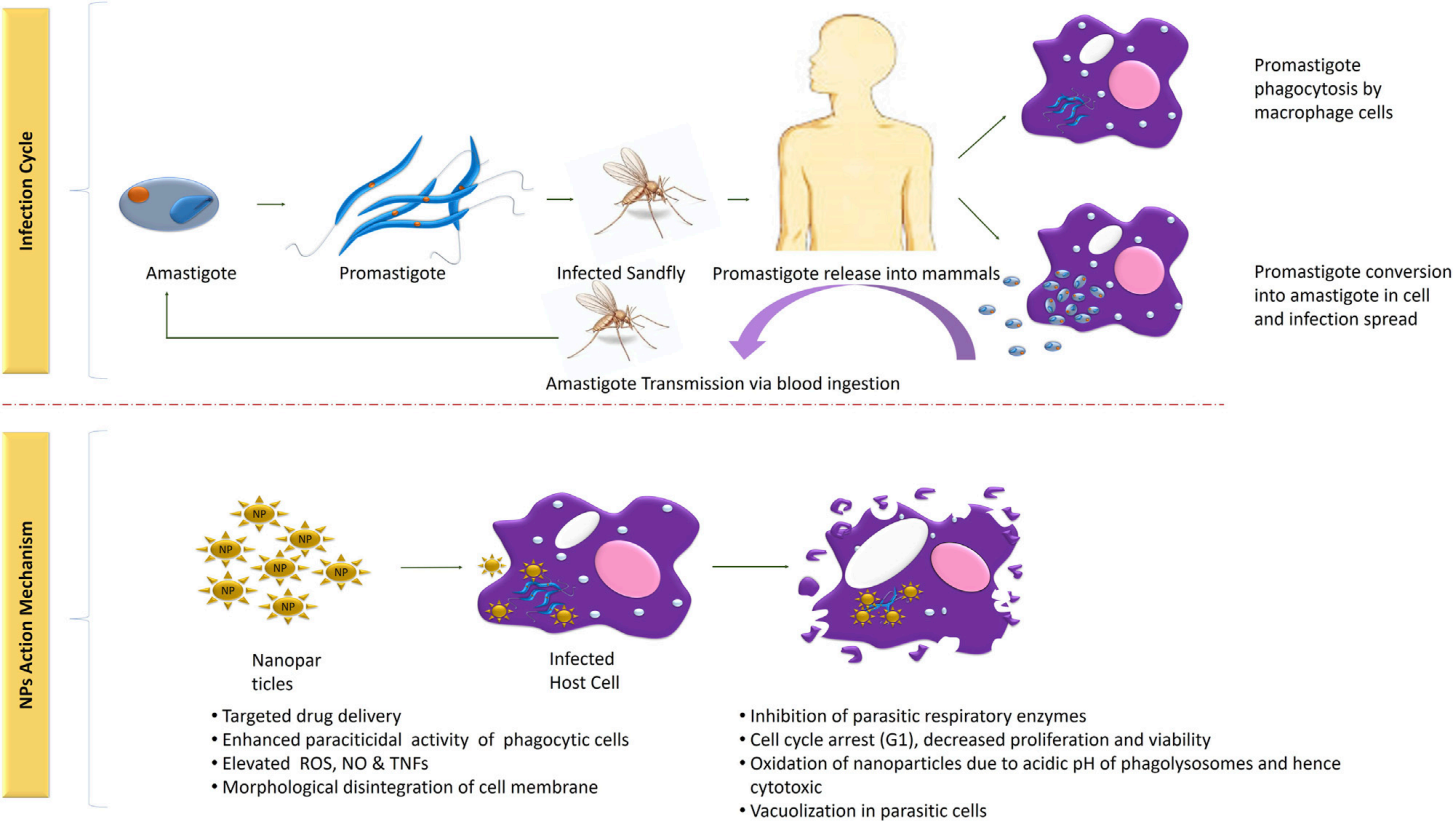
Infection cycle and mechanism of action of NPs in the treatment of *Leishmania*.

The life cycle of *Leishmania* parasite begins with the bite of infected female phlebotomine sandflies and is completed in two different morphological forms, that is, promastigote and amastigote. Flagellated metacyclic promastigote, formed in the infected sandflies firstly enter into the macrophages via phagocytosis and formed phagosome. Resultant phagosome enter into the stages of maturation (membrane transformation) and formed a new structure: the parasitophorous vacuole. In this organelle, promastigotes metamorphose into amastigote, followed by its multiplication until explosion of the cells of the macrophage system (4–6°days) spreads infection further. The parasite either initiates infection superficial cells or visceral cell depending on its tropism characteristics. The life cycle of *Leishmania* into the host is completed when another uninfected sandfly sucks blood as a source of meal. The sensitivity of infection is based on the sandfly species, ecology, epidemiology, and pathogenicity (Bañuls et al., 2007). Further, detailed description of its infection period and transmission is clearly shown in Figure 1.

## Existing Therapeutic Approaches and Limitations

Leishmaniasis is one of the most important NTDs coupled with various adverse as well as life-threating factors, including substantial morbidity, early death, and long-term infirmity. Treatment comprises control of disease spreading and use of existing parameters, while the currently used therapies including chemical drugs necessitate long-duration therapy and low efficiency with numerous toxic effects. Although no relevant therapies have been developed to prevent the infection which is extensively spread among human population, only few prevention methods are available (Gharirvand Eskandari et al., 2020). Among them, some kind of clinically approved drugs are found to treat this endemic disease, including meglumine antimoniate (glucatime), sodium stibogluconate (pentostan), amphotericin B, and miltefosine*.* However, the excessive use of these chemotherapeutic sources is associated with antagonistic effects (Ghobakhloo et al., 2016). This has led to the search of some natural methods to treat leishmaniasis.

### Chemical-Based Drugs for the Treatment of Leishmaniasis

Since last several years, various kinds of pharmaceutical drugs including amphotericin B, pentamidine, miltefosine, and paromomycin were involved in the treatment of leishmaniasis. None of the clinically approved drugs could be deliberated as the ultimate source of treatment due to their time-taking process and high toxicity combined with severe adversative effects. In addition, the most often used medicines do not eradicate the parasites entirely from all infected entities (De Menezes et al., 2015). Further, applications of some of these medicines with their limitations are described below:

• Pentavalent antimonials can be administrated by the intravenous, intramuscular, and intralymphatic routes with the optimum dosage of 20 mg/kg/day (28–30 days) and exhibited 35–95% potentiality. Continuous and excessive use of this drug causes toxicity like nephrotoxicity, hepatotoxicity, severe cardiotoxicity, and pancreatitis (De Menezes et al., 2015)*.*
• Oral administration of miltefosine not only showed inhibitory effects on the growth of *Leishmania* but also affected adversely and created severe infecting symptoms comprising nephrotoxicity, teratogenicity, vomiting and diarrhea, and hepatotoxicity (Sundar et al., 2011).
• Paromomycin, also being used as a therapeutic agent to treat leishmaniasis, reported to show some toxic effects during its treatment phase, like severe nephrotoxicity, hepatotoxicity, and ototoxicity (Jhingran et al., 2009).
• Pentamidine with the prime dosage of 3 mg/kg/day can potentially involve in the retardation of *Leishmania* growth with some severe antagonistic effects such as hypotension, elevated rate of hyperglycemia, tachycardia, pancreatic damage, and electrocardiographic changes (De Menezes et al., 2015).

The existing chemotherapies have a list of short comings comprising high cost, higher toxicity, and acquired resistance toward parasitic strain, and other side effects during their prevention mechanism insisted scientists and medical practitioners to evolve a new therapeutic system to treat NTDs. During the last decades, green therapies involving plant extracts, bioactive compounds, and secondary metabolites derived from particular plant species and different kinds of NPs synthesized using plant extract become promising as well as safer prevention therapies.

### Natural Methods

From ancient times, plant-based traditional methods are being used in the therapeutics against various infectious ailments. Currently, plant extract and particular bioactive compound extracted from plants are either directly used as a therapeutic source or as derived herbal drugs for the treatment of leishmaniasis as well as other microbial infection (Oryan, 2015).

#### Involvement of Plant Extracts and Plant-Derived Secondary Metabolites

The consumption of herbal drugs derived from plants is being used from centuries as a prevention source for NTDs as well as other diseases including bacterial (and their vectors), helminth (and their vectors), fungal, ectoparasitic, protozoan (and their vectors), and viral infections (and their vectors). The use of medicinal plants becomes more advantageous over other chemotherapies due to their nontoxic, environment-friendly, and cost-effective properties. Further natural compounds obtained from plants are considered as a reliable therapeutic source to treat leishmaniasis (Cheuka et al., 2017).

*Ageratum conyzoides*, *Bidens pilosa*, and *Eugenia uniflora* showed efficient leishmanicidal effects. *Bidens pilosa* (root) has been reported for its antileishmanial properties (against *L. amazonensis*, promastigote) with the least IC_50_ value (1.5 μg/ml) as compared to other plant species (Table 1). Essential oils from *Eugenia uniflorae* potentially inhibit the growth of both the parasitic forms, that is, promastigote and amastigote, of *L. amazonensis*, and *Ageratum conyzoides* has been reported to treat infection caused by *L. donovani* (amastigote form) (Silveira et al., 2021). E-caryophyllene, the main component of *Melampodium divaricatum* and *Casearia sylvestris* essential oil, has been reported for its promising antileishmanial response against *L. amazonensis* (IC_50_ values of 10.7, 10.7, and 14.0 μg/ml) (Moreira et al., 2019). Moreover, 1,8-cineole, α-pinene, and p-cymene active constituents of *Protium altsonii* and *P. hebetatum* (Burseraceae) exhibited dose-dependent amastigote inhibition with IC_50_ of 48.4, 37, and 46 μg/ml, respectively (Santana et al., 2020). Butanol fraction of *K. odoratissima* with 154.1 μg/ml IC_50_ value showed antileishmanial properties against *L. major* promastigote and amastigote (Mirzaei et al., 2020).

**TABLE 1:** Role of plant-based natural products in the treatment of leishmaniasis.

S. no	Plant used	Plant part used for extract preparation	Bioactive compound involved	Mode of study and optimum dosages	Organism tested		Structural formula	Mechanism of action	References
1	*Baccharis uncinella* (groundsel)	Leaves	Ursolic acid	*In vivo* 1.0 mg/kg or 2.0 mg/kg (body weight)	*L. infantum*		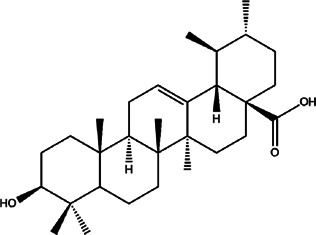	Treatment with ursolic acid causes a remarkable reduction in liver as well as splenic parasitism	Jesus et al. (2017)
2	*Allium sativum* (garlic)	Bulb	Allicin	*In vitro* and *in vivo* 50 μM for *in vitro* studies	*L. major*		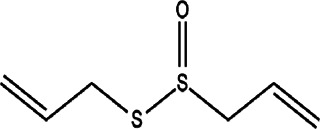	-	Metwally et al. (2016)
3	*Eremurus persicus* (desert candles)	Root extract	Aloesaponol III 8-methyl ether	*In vitro* IC_50_ 73 μg/ml	*L. infantum*		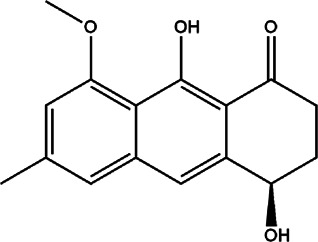	After treatment with isolated compound, mitochondrial potential and few structural alterations in the promastigote form of tested organism were observed	Rossi et al. (2017)
4	*Olea europaea* (wild olive, Indian olive, and brown olive)	Air-dried, pulverized leaves	Oleuropein	*In vitro* and *in vivo* 128.4 μM (69.4 μg/ml), for *in vitro* studies	*L. donovani*		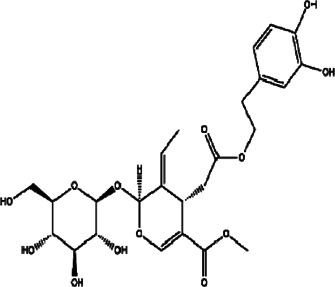	Oleuropein responsible to raise ROS production, upregulation of host antioxidant enzymes, and downregulation of other enzymes of the parasite. Furthermore, *in vivo* model delayed-type hypersensitivity and elevation of IgG2a/IgG1 ratio (leishmania-specific) were observed	Kyriazis et al. (2016);Sharma et al. (2019)
5	*Zingiber zerumbet* (awapuhi and bitter ginger)	Fresh rhizome	Zerumbone	*In vitro* 10 μM	*L. donovani*		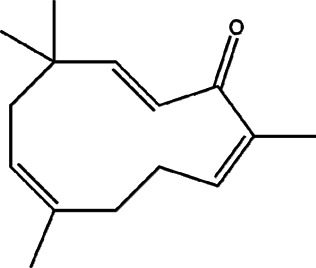	Zerumbone extracted from *Zingiber zerumbet* causes apoptosis in promastigotes by affecting ROS production coupled with reduction of intracellular amastigotes in infected macrophages	Mukherjee et al. (2016)
6	*Morinda lucida* (brimstone tree)	-	Molucidin	*In vitro* IC_50_ 4.24 μM for *Leishmania hertigi* and anti-010 activity with MIC of 4.167 μM	*L. hertigi* and field strain-010		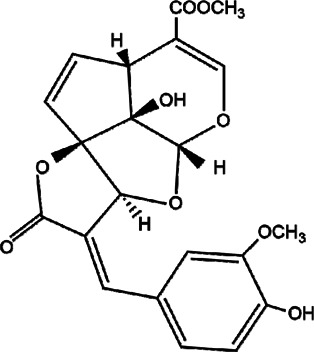	Molucidin: Normal cells have single set of nucleus and kinetoplast, that is, 1N/1K, but molucidin stimulates two different sets of kinetoplast and nucleus in the cells of parasite. After division of both the sets, this compound obstructs the cytokinesis and causes cell cycle arrest which leads to death of parasites	Amoa-Bosompem et al. (2016);Sharma et al. (2019)
7	*Artemisia annua* (sweet annie, annual mugwort, sweet sagewort, or annual wormwood)	-	Artemisinin	*In vivo* and *in vitro* 100 μg/ml for *in vivo* studies	*L. major*		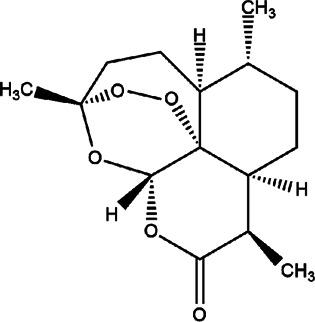	-	Ghaffarifar et al. (2015); Sharma et al. (2019)
8	*Hypericum Carinatum* (St John’s wort)	Flowering aerial parts	Cariphenone A (1), isouliginosin B (2), and uliginosin B (3)	*In vitro* IC_50_ values of 10.5, 17.5, and 11.3 µM for compound 1, 2, and 3, respectively.	*L. amazonensis*		1 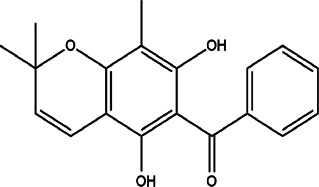 2 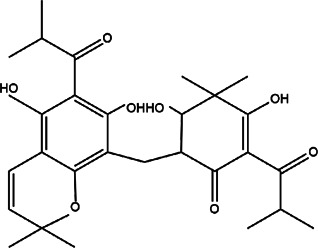 3 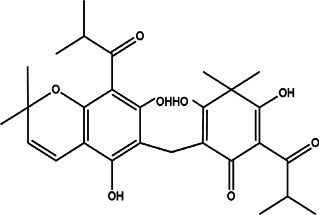	Inhibition of parasites mediated by oxidative stress (ROS production) and alteration in mitochondrial potential-like hyperpolarization condition	Dagnino et al. (2018)
9	*Euphorbia peplus* (radium weed)	Peplus aerial parts	Simiarenol	*In vitro* IC_50_ values of 20.24, 34.87, and 32.05 μg/ml	*L. donovani*		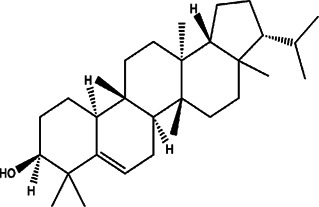	-	Moawad et al. (2016)
10	*Strychnos pseudoquina*	-	Strychnobiflavone	*In vitro* 5.4 and 18.9 μM	*L. amazonensis*		-	Mechanism of action will be allied with alteration in mitochondrial membrane potential in parasitic cells	Lage et al., 2015
11	*Melampodium divaricatum* (butter daisy) and *Casearia sylvestris*	Essential oils ( aerial parts of *Melampodium divaricatum* and leaves of *Casearia sylvestris*	E-caryophyllene (22.2%), germacrene D (19.6%), and bicyclogermacrene (12.2%)	*In vitro* 24.2, 29.8, and 49.9 μg/ml	*L. amazonensis*		1 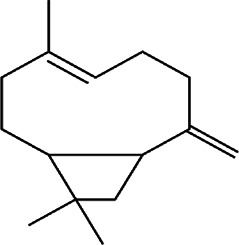 2 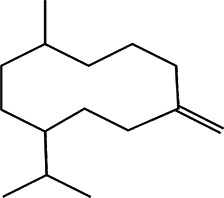 3 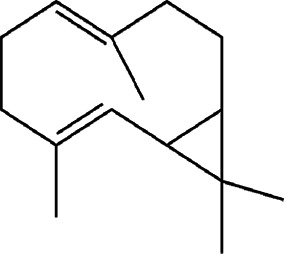	-	Moreira et al. (2019)
12	*Handroanthus* species	-	Lapachol	*In vitro* and *in vivo* (IC_50_ = 79.84 ± 9.10 μM, SI = 42.65) for *L. amazonensis* and (IC_50_ = 135.79 ± 33.04 μM, SI = 25.08) for *L. infantum* 25 mg/kg for *in vivo* model	*L. infantum* and *L. amazonensis*		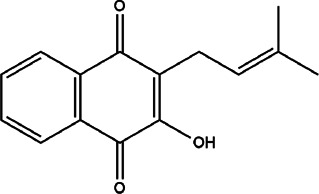	In the *in vivo* model, lapachol will be able to reduce the parasitic load in the spleen, liver, and skin lesions	Araújo et al. (2019)
13	*Ifloga spicata* (*I. spicata*) (alj al anza, alj al ghazal, and hasaj)	Whole plant (leaves, flowers, stem, and roots)	3,4-Dihydroxybenzoate (compound 1) and benzoate (compound 2)	*In vitro* LD_50_ values of 10.40 ± 0.09 and 14.11 ± 0.11 μg/ml for compound 1 and compound 2, respectively.	*L. tropica*		1 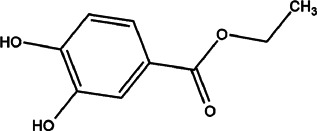 2 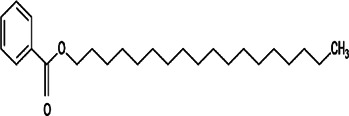	Both the isolated compounds showed great affinity with gp63 (leishmanolysin) receptor of leishmania parasite. Binding of these compounds with their receptors resulted in the smooth entry of parasites into the cells, and after injection, it binds with DNA and causes apoptosis	Shah et al. (2019)
14	*Protium altsonii* (PaEO) and *P. hebetatum* (PhEO)	-	Essential oil	*In vitro* PaEO IC_50_ were 14.8 μg/ml and 7.8 μg/ml and PhEO IC_50_ were 0.46 μg/ml and 30.5 μg/ml	*L. amazonensis*		-	Mitochondrial membrane potential associated with NO production could be an effective mechanism of leishmaniasis	Santana et al. (2020)
15	*Artemisia aucheri*	Whole plant extract	*-*	*In Vitro* and *in vivo* IC_50_ 90 μg/ml	*L. major*		-	-	KarimiPourSaryazdi et al. (2020)
16	*Clerodendrum myricoides* (blue-flowered tinder wood) and *Salvadora persica* (arak, jhak, pīlu, *Salvadora indica*, toothbrush tree, mustard tree)	Aqueous extract of stem	-	*In vitro* MIC = 625 μg/ml	*L. major*		-	-	Maina et al.(2020)
17	*Croton blanchetianus* Baill	Ethanolic extract	-	*In vitro* IC_50_ values of 208.6 and 8.8 μg/ml for *Leishmania infantum* and IC50 values of 73.6 and 3.1 μg/ml for *Leishmania amazonensis* promastigotes and amastigotes	*L. amazonensis* and *L. infantum*		-	Ethanolic extract of *Croton blanchetianus* targets a significant depolarization of mitochondrial membrane potential and leads to mitochondrial dysfunction	Pereira et al. (2020)
18	*Prunus armeniaca* (Armenian plum)	Leaf extract	1, 2-benzenedicarboxylic acid and diisooctyl ester	*In vitro* anti-promastigote activity with IC_50_ 11.48 ± 0.82 μg/ml and anti-amastigotes activity with IC50 21.03 ± 0.98 μg/ml	*L. tropica*		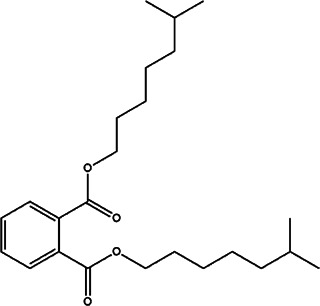	-	Shaheen et al. (2020)
19	*Urtica dioica* (common nettle, stinging nettle)	Aqueous extract	-	*In vivo and in vitro* 3,500 and 6,000 μg/ml for promastigotes and amastigotes, respectively.	*L. major*		-	It proficiently killed the amastigote form of *L. major*; additionally, remarkable reduction of parasite load, skin lesion size, and IL-4, and significant increase of NO and IFN-γ were observed	Badirzadeh et al. (2020)
20	*Piper marginatum* (cake bush, anesi wiwiri, marigold pepper)	Leaves (ethanolic extract)	3,4-Methylenedioxypropiophenone	*In vivo*	*L. amazonensis*		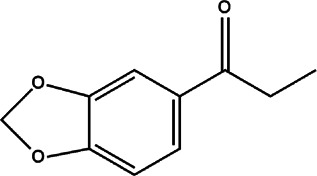	-	Macêdo et al. (2020)
21	*Kelussia odoratissima* (kelus celery and wild celery)	Dried leaves (butanol fraction)	-	*In vitro* half (IC_50_) 264.1 and 154.1 μg/ml for promastigotes and amastigotes, respectively.	*L. major*			-	Mirzaei et al. (2020)
22	*Tabernaemontana coronaria* (milkwood)	Dried powder of stem bark	Voacamine	*In vivo* IC_50_ value was found to be 14.702 ± 0.101 mM	*L. donovani*		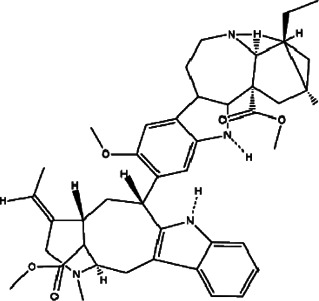	Voacamine supresses the relaxation potential of LdTop1B (*L. donovani* toposoisomerase IB) and makes the clevable complex steady	Chowdhury et al. (2017)
23	*Picramnia gracilis* (bitterbush)	Powder of dried leaves	5,3′-hydroxy-7,4′- dimethoxyflavanone	*In vitro* and *in vivo* EC_50_ 17.0 + 2.8 mg/ml, 53.7 µM for *in vitro* and 2 mg/kg/day for *in vivo* studies	*L. braziliensis*		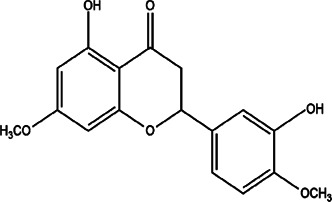	-	Robledo et al. (2015)
24	*Lindera aggregate* (spice bush)	Leaves/bark	Boldine	*In vitro* 600 μg/ml	*L. amazonensis*		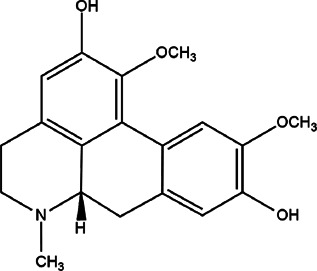	-	Salama et al. (2017)
25	*Hypericum andinum*	Dried and powered materials of aerial parts	Uliginosin B	*In vitro* (IC_50_) of 36.1 g/ml	*L. amazonensis*		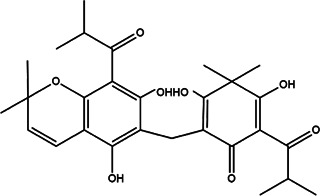	-	Dagnino et al. (2015)
26	*Amphilophium crucigerum* (monkey’s comb)	Aerial parts	Ipolamiide	*In vitro* IC_50_ = 100 µM	*L. amazonensis*		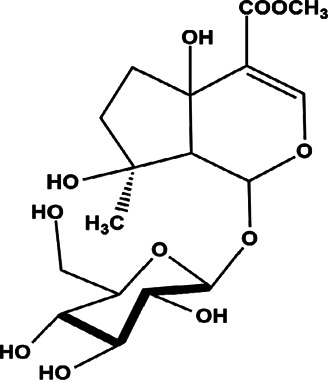	-	Vendruscolo et al. (2018)
27	*Valeriana jatamansi* (Indian Valerian or Tagar)	Rhizomes	Valepotriates	*In vitro* IC_50_ = 2.96 µM	*L. major*		-	-	Glaser et al. (2015)
28	*Nymphoides indica* (banana plant, robust marshwort, and water snowflake)	Leaves	3-O-methylquercetin-7-O-β-glucoside	*In vitro* IC_50_ 32 μM	*L. infantum*		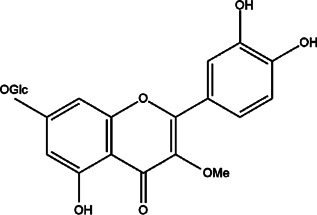	-	Amin et al. (2016)
29	*Vitex grandifolia* (black plum, chocolate and berry tree)	Air-dried leaves	Bartioside	*In vitro* IC_50_ 27.51 µM	*L. donovani*		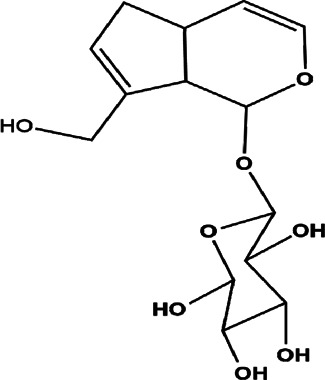	-	Bello et al. (2018)
30	*Scrophularia syriaca* (figworts)	Aerial parts	6-O-a-lrhamnopyranosylcatalpol	*In vitro* EC_50_ 100 µM	*L. major*		-	-	Alkhaldi et al. (2020)
**Section 2: Natural drugs for treating leishmaniasis**									
**S. no**	**Compound (s)**	**Company/originator**	**Country**	**Year**		**Mode of studies**	**Probable mechanism involved**	**Patent and IPC**	**Ref.**
31	Ethyl 3-(2- chloroacetamido) benzoate, dihydroquercetin, and bisabolol	Auclair et al., AC Bioscience SA	Switzerland	2019		*In vitro* and *in vivo* studies	Inhibition of some important parasitic enzymes with tryparedoxin peroxidase and tubulin	WO2019043212 and A61K A61P	Hajaji et al. (2018)
32	Diterpenoid membranolides	Baker et al., the University of South Florida	United States of America	2016		*In vitro*	Impedes lipid synthesis	US2016003O388 and A61K	Baker et al. (2016)
33	Withaferin-A and miltefosine	Maurya et al., the University of Hyderabad	India	2017		*In vitro* and *in vivo* studies	Inhibits pteridine reductase-1 enzyme, and phosphatidylcholine synthesis, and cytochrome c oxidase	WO2017046778and A61K A61P	Maurya and Chandrasekaran (2017)

#### Role of Plant-Based Nanoparticles in the Treatment of Leishmaniasis

Methods in controlling infectious diseases have modernized translational sciences to develop a better controlling method for infectious diseases. The field of nanomedicine has shown enormous potential in developing highly sensitive diagnostic tools with excellent drug delivery properties. Recently, nanoparticle-conjugated drugs have increasingly been studied as an alternative, cost-effective therapy with increased effectiveness. However, toxicity is a major barrier that needs to be encountered. Several reports have shown the effective antimicrobial activities of various metal/metal oxide nanoparticles as well as against the *Leishmania* causing organism through their wide surface area and unique properties.

Nanoparticles synthesized using crude as well as various solvent-fractionated extracts of medically important plants are considered as efficient agents for the delivery of specific phytoconstituents into the cells. Keeping in view the effective antimicrobial activities of silver metal, silver/silver oxide NPs have been synthesized using a variety of medicinally important plant species, including *Mentha arvensis* L., *Ficus benghalensis*, *Cuminum cyminum*, *Moringa oleifera*, *Silybum marianum*, and *Sechium edule*, at a dosage of 10, 300, 0.5, 246, and 51.88 μg/ml tested against *L. tropica*, *L. donovani*, *L. tropica*, *L. major*, *L. tropica*, and *Leishmania donovani*, respectively (Baranwal et al., 2018; El-Khadragy et al., 2018; Hameed et al., 2019; Ismail et al., 2019; Bagirova et al., 2020; Javed et al., 2020). Gold and silver bimetallic NPs synthesized by using medically important plants have also been reported to be used for the prevention of this disease (Alti et al., 2020). However, *Cannabis sativa*–based Au-NPs accomplished virtuous antileishmanial activity against amastigote forms (IC_50_: 171·00 ± 2·28 μg/ml) (Hameed et al., 2020). 7, 8-dihydroxyflavone, a type of flavonoid found abundantly in plants used to produce gold nanoparticles, has also been reported to preclude leishmaniasis (Prasanna et al., 2020).

Zno-NPs were also reported to show dose-dependent cytotoxicity against *L. tropica* (IC_50_: 8.30 μg/ml) (Iqbal et al., 2019). Rod-shaped zinc oxide NPs produced by using *Lilium ledebourii* tuber extract potentially inhibited the growth of *L. major* with the IC_50_ value of 0.001 mg ml^−1^ (Khatami et al., 2020). Saleh (2019) also concluded that green TiO_2_ nanoparticles have shown effective roles to counter noxiousness of *Leishmania tropica* in male rats. Hematite (Fe_2_O_3_) NPs fabricated with the *Rhus punjabensis* extract played an efficient role in the treatment of leishmaniasis (Naz et al., 2019). Khalil et al. (2020) prepared lead oxide NPs (PbO-NPs) by green route using aqueous leaf extracts of *Sageretia thea*. The experimental data showed that PbO-NPs were significantly active in arresting the growth of promastigote and amastigote forms of *Leishmania tropica*, with 14.7 μg/ml and 11.95 μg/ml IC_50_ values, respectively.

Plant-mediated (*Trigonella foenum-graecum*) iron oxide nanoparticles have been reported to exhibit significant inhibitory effects on *L. tropica* (Ain et al., 2019). Further, Abbasi et al. (2019) also stated the antileishmanial efficacy of Nio-NPs fabricated by using *Geranium wallichianum* against *L. tropica*.

Besides, the nanostructured drug delivery system was also reported in ameliorate NTDs including leishmaniasis. Furthermore, crude plant extracts and precise phytoconstituents obtained from plant which is involved in the prevention mechanism were also loaded in the nanostructured drug delivery system and used as a therapeutic source to cure leishmaniasis, and the mechanism is depicted below:

• Liposome NPs consisting of phospholipids are assisted as a transport system for the delivery of hydrophilic as well as lipophilic pharmaceutical drugs (Momeni et al., 2013). They provide improved pharmacokinetic assets along with target diligence which offers a foremost advantage (Kaye and Scott, 2011). Liposome can spear the macrophages through phagocytosis and offers direct delivery of the drugs at their targeted sites. Different drug formulations including AmB colloidal formulations, liposomal AmB, and AmB lipid network can overwhelm the toxic effects of conventional drugs (Moreno et al., 2015). Liposome-encapsulated *Curcuma longa* and *Combretum leprosum* extracts were also reported for their antileishmanial properties (Aditya et al., 2012; Barros et al., 2013).
• Beta-lapachone extracted from Lapacho tree with the use of lecithin-chitosan NP encapsulation method has been reported in the treatment of leishmaniasis (Moreno et al., 2015).
• 8-hydroxyquinoline with the polymeric micelle encapsulation method has been used to treat *Leishmania* (Duarte et al., 2016). Berberin, an isoquinoline alkaloid extracted from medicinal plants, has been reported to possess various biologic properties, including antileishmanial properties. A previous study addressed the preparation of BER-loaded liposomes with the aim to prevent its rapid liver metabolism and improve the drug selective delivery to the infected organs in visceral leishmaniasis (VL) (Calvo et al., 2020).

As per the literature survey, plant-based nanoparticles contribute efficient roles in the treatment of leishmaniasis as compared to other existing practices. Phytosynthesized NPs revealed an identical effect on the inhibition of parasitic growth at a comparatively lesser concentration than the prescribed dose of Amp B to cure this disease. Additionally, bimetallic nanoparticles including Au−Ag, Zn−Ag, and Ti−Ag were synthesized using the green approach and proficiently used as a therapeutic source to treat leishmaniasis (Alti et al., 2020). NPs are preferred over other therapeutic sources to treat this dreaded disease because of their nontoxic, harmless, and efficient delivery system for vaccine*.* Currently, with the advancement of nanosciences, there is a new method of synthesizing vaccines using NPs as carriers of antigen preparation. Solid lipid nanoparticles can assist as an effective tool to produce leishmanial vaccine (Saljoughian et al., 2013). However, any kind of NP-based vaccine is not accessible, and it needs more consideration.

## Restorative Mechanism of Nanoformulations Against Leishmaniasis

*Leishmania* sp. are protozoal parasites which result in cutaneous and visceral leishmaniasis. Different clinical studies exhibit the development of self-curable to detrimental conditions, depending upon the immune responses triggered by the affected host (Noormehr et al., 2018). Chemotherapy with pentavalent antimonials (like sodium stibogluconate or meglumine antimoniate) and other antileishmanial drugs (amphotericin B, fluconazole, pentamidine, and miltefosine) are optimal for leishmanial therapy. However, due to adverse effects, high cost, difficult infusion routes, low cure, and increasing resistance are of significant concern in developing more efficient ways in leishmaniasis therapy. Moreover, the efficacy of the drug used in the treatment also varies for different leishmanial sp. (Noormehr et al., 2018; Alti et al., 2020; Calvo et al., 2020). In self-treatment, the innate immune cells (phagocytes) detect and engulf the causal agents, which induces *Leishmania* assassination by producing reactive oxygen species, nitric oxide, and tumor necrosis factors (Olekhnovitch et al., 2014). After innate immune responses, respective activation and production of CD8^+^, NK, and IFN cells by TH1 immunity results in killing of *Leishmania* parasites (Noormehr et al., 2018). In susceptible conditions, the defense system fails to overcome infections, and follows incorrect TH2 immune responses along with antibody response, which is the key factor to generate new ways of parasite elimination. Metal nanoparticles inhibit proliferation and viability of infected cells, which is contingent with the NP strength and time of exposure (Rosas-Hernández et al., 2009; Fanti et al., 2018).

Several *in vitro* as well as *in vivo* findings suggest leishmanicidal effects of bio–Ag-NPs by direct (exclusive of inflammatory mediators) or indirect (immunomodulatory) mechanisms (Fanti et al., 2018; Calvo et al., 2020). In the direct method, metal-NPs kill the parasitic cells by causing vacuolation inside parasites and damage to the cellular membrane without generating immunomodulatory intermediaries, that is, reactive oxygen species (ROS), nitric oxide (NO), and apoptotic and necrotic factors (Fanti et al., 2018). In situations when *Leishmania* parasites override the oxidative burst inside phagocytic cells and reside in phagolysosomes, nanoformulations assist site-specific delivery and accumulation of drugs, which is responsible for parasite killing (Shoaib Sarwar et al., 2020). According to Fanti et al. (2018), Ag-NPs after diffusion through the cellular membranes get oxidized due to acidic conditions within the phagolysosomes, and eventually, the release of free Ag + ions causes parasite assassination.

On the other hand, the indirect method involves immunomodulatory response generation at infection sites. Other ways to provide leishmanicidal effects are through activating immune response mediators in which the cell viability and proliferation get declined as an effect of metallic nanoparticles. NPs basically induce different morphological abrasions such as distorted membranal integrity, cytotoxicity, mitochondrial destruction, cell cycle arrest (G1), increased/decreased ROS and NO generation, affected enzymatic activities, and release of apoptotic or necrotic factors (Park et al., 2010; Kruszewski et al., 2011; Zahir et al., 2015). As a result of mitochondrial disintegration, ATP generation gets influenced, which causes cytotoxic effects, and ultimately affects the infection growth (AshaRani et al., 2009). Moreover, NP exposure exhibits decreased parasitic load and reduction in an essential parasitic enzyme trypanothione reductase system (Fanti et al., 2018).

## Conclusion and Future Prospective

Chemotherapy due to lack of effective therapies till date has become the only choice in treating leishmaniasis, as these therapies exhibit higher toxicity levels, treatment cost, and resistance development against leishmanial parasites, and encourage other side effects. In addition, it is evident that the efficiency of drugs varies from species to species due to leishmanial antigen variants and different immunological responses against the drug. To overcome these challenges, biogenic nanomaterials being nontoxic, bio-compatible, cost effective, and having high targeted drug-loading potentials have been indicated as beneficial alternatives to formulate nanovaccines. Targeted drug delivery barriers can be conquered by using nanoformulations for enhanced parasiticidal proficiencies. Also, various studies have demonstrated leishmanicidal activities of plant-derived natural compounds (such as berberine, 7, 8-dihydroxyflavone, E-caryophyllene, essential oil constituents, α-terpineol, glycosides, tannins, and anthraquinone flavonoids), which can further integrate beneficial outcomes. Besides, most of the studies conducted on leishmanicidal activities revealed only the basic outcomes like assessment of the effect of test drugs (crude extract, isolated bioactive compounds, essential oil, and purified fraction) on the parasite growth. Few of them identify the proper formulation as well as the effect on the promastigote stage, found in the sandflies (vector). As widely conferred in the literature, plants possess a variety of bioactive compounds, and most of them have been reported for their pharmaceutical properties. Thus, the standardization may conclude the identification of particular compound responsible for leishmanicidal activities. Biosynthesized nanoparticles majorly eliminate the infection either by triggering the immunomodulatory response of the host or sometimes directly by resulting in vacuolization of parasitic cells, leading to parasite killing. Nanovaccines are a relatively new concept in treating *Leishmania* although no vaccine is yet available, but studies are ongoing to find efficient nanovaccines. Although nanotechnology has provided a hope toward improved and successful eradication of neglected tropical diseases, the accurate molecular mechanism responsible still needs thorough transparency to bring utmost benefits.
