# Supplementary material for: Green Approach: ‘‘A Forwarding Step for Curing Leishmaniasis—A Neglected Tropical Disease’’
Source: Front Mol Biosci. 2021 May 28;8:655584. doi: 10.3389/fmolb.2021.655584 (PMC8193676; doi:10.3389/fmolb.2021.655584)
Supplement: Supplementary file 1 [file Table1.DOCX]

| S.no. | Plant used | Plant part used for extract preparation | Bioactive compound involved | Mode of study & Optimum dosages | Organism tested | Structural formula | Mechanism of action | References |
| --- | --- | --- | --- | --- | --- | --- | --- | --- |
| 1 | *Baccharis uncinella*  *(*groundsel) | Leaves | Ursolic acid | *In vivo*  1.0 mg/kg or 2.0 mg/kg (body weight) | *L. infantum* | 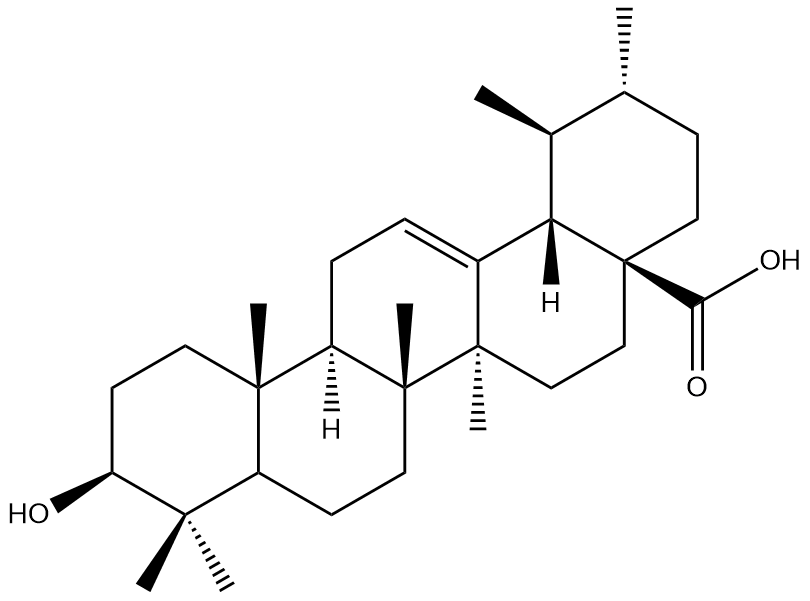 | Treatment with ursolic acid causes a remarkable reduction in liver as well as splenic parasitism. | (JA et al., 2017) |
| 2 | *Allium sativum*  (garlic) | Bulb | Allicin | *In vitro & In vivo*  50 μM for in vitro studies | L. major | 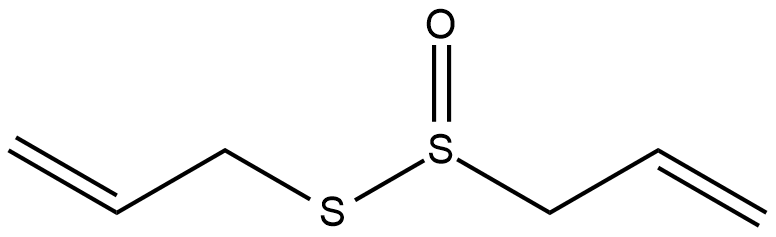 | - | (Metwally et al., 2016) |
| 3 | *Eremurus persicus*  (desert candles) | Root extract | Aloesaponol III 8-  methyl  ether | *In vitro*  IC_50_ 73 µg/mL | *L. infantum* | 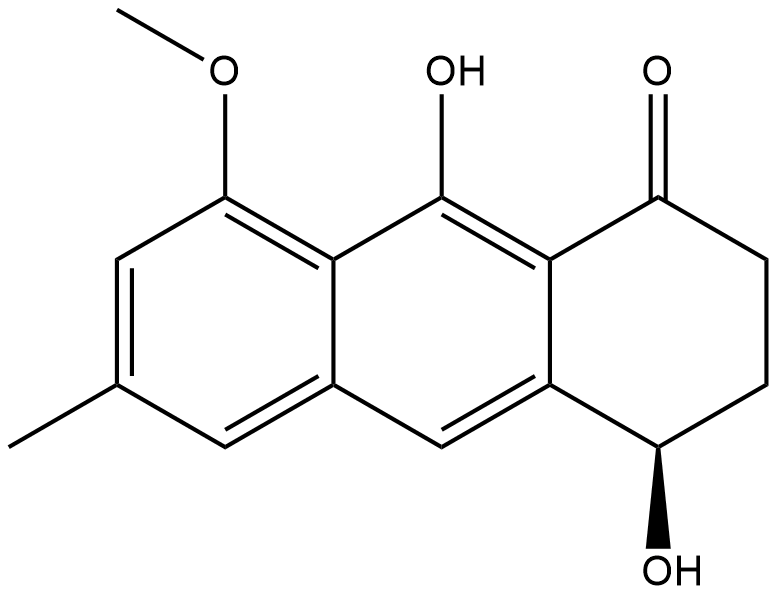 | After treatment with isolated compound mitochondrial potential and few structural alterations in the promastigote form of tested organism were observed. | (Rossi et al., 2017) |
| 4 | *Olea europaea*  (wild olive, Indian olive brown  olive,) | Air-dried, pulverized leaves | Oleuropein | *In vitro & In vivo*  128.4 μM (69.4 μg/ml), for *In vitro* studies | *L. donovani* | 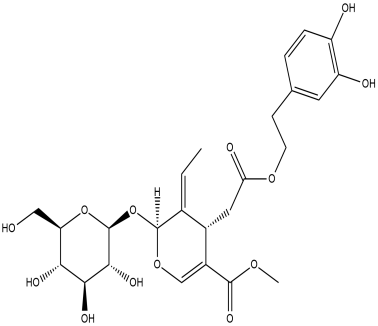 | Oleuropein responsible to raise ROS production, upregulation of host antioxidant enzymes as well downregulation of other enzymes of parasite. Furthermore, in *in vivo* model delayed-type hypersensitivity and elevation of IgG2a/IgG1 ratio (leishmania specific) was observed. | (Kyriazis et al., 2016; Sharma et al., 2019) |
| 5 | *Zingiber zerumbet*  (awapuhi, bitter, ginger) | Fresh rhizome | Zerumbone | *In vitro*  10 μM | L. donovani | 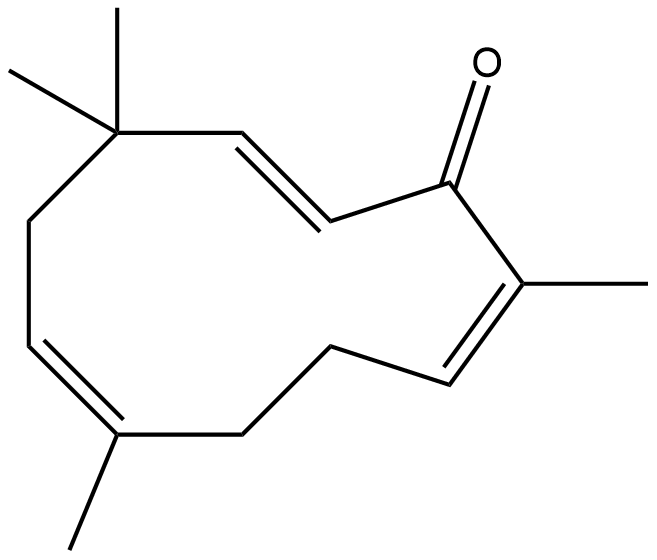 | Zerumbone, extracted from *Zingiber zerumbet* causes apoptosis in promastigotes by affecting ROS production coupled with reduction of intracellular amastigotes in infected macrophages. | (Mukherjee et al., 2016) |
| 6 | *Morinda lucida*  (brimstone tree) | - | Molucidin | *In vitro*  IC_50_ 4.24 μM for  *Leishmania hertigi*.and anti-010 activity with MIC of 4.167 μM | *L. hertigi* & field strain-010 | 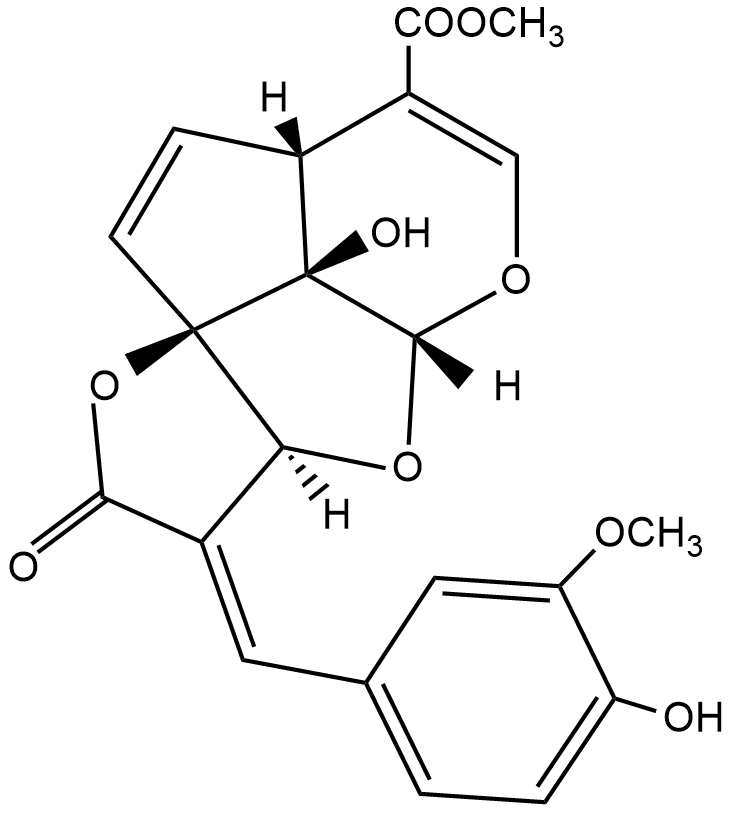 | Molucidin: Normal cells have  single set of nucleus and  kinetoplast i.e. 1N/1K, but  Molucidin stimulates two different sets of kinetoplast and nucleus in the cells of parasite. After division of both the sets this compound obstruct the cytokinesis causes cell cycle arrest which leads to death of parasites. | (Amoa-Bosompem et al., 2016; Sharma et al., 2019) |
| 7 | *Artemisia annua*  (sweet annie, annual mugwort sweet,sagewort or annual wormwood) | - | Artemisinin | *In vivo & In vitro*  100 μg/ml for  *In vivo* studies | *L.major* | 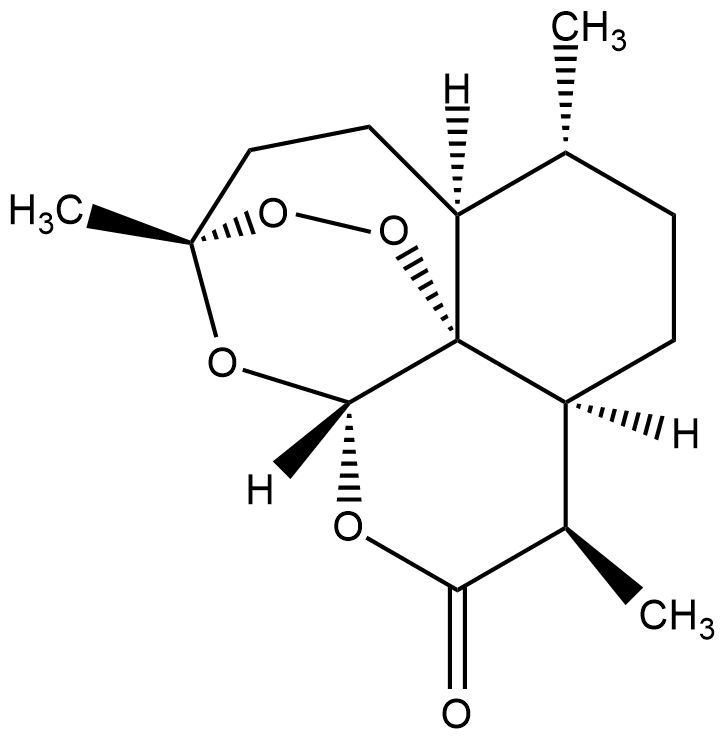 | - | (Ghaffarifar et al., 2015; Sharma et al., 2019) |
| 8 | *Hypericum*  *Carinatum*  (st John's  wort) | Flowering aerial parts | cariphenone A (1), isouliginosin B (2) and uliginosin B (3) | *In vitro*  IC_50_ values of 10.5, 17.5 and 11.3 µM for compound 1,2,3 respectively | *L. amazonensis* | 1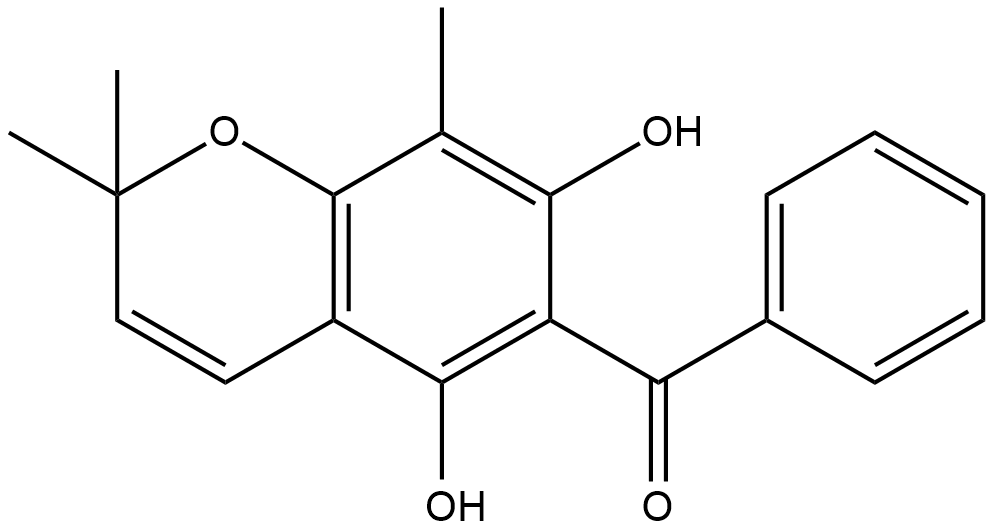  2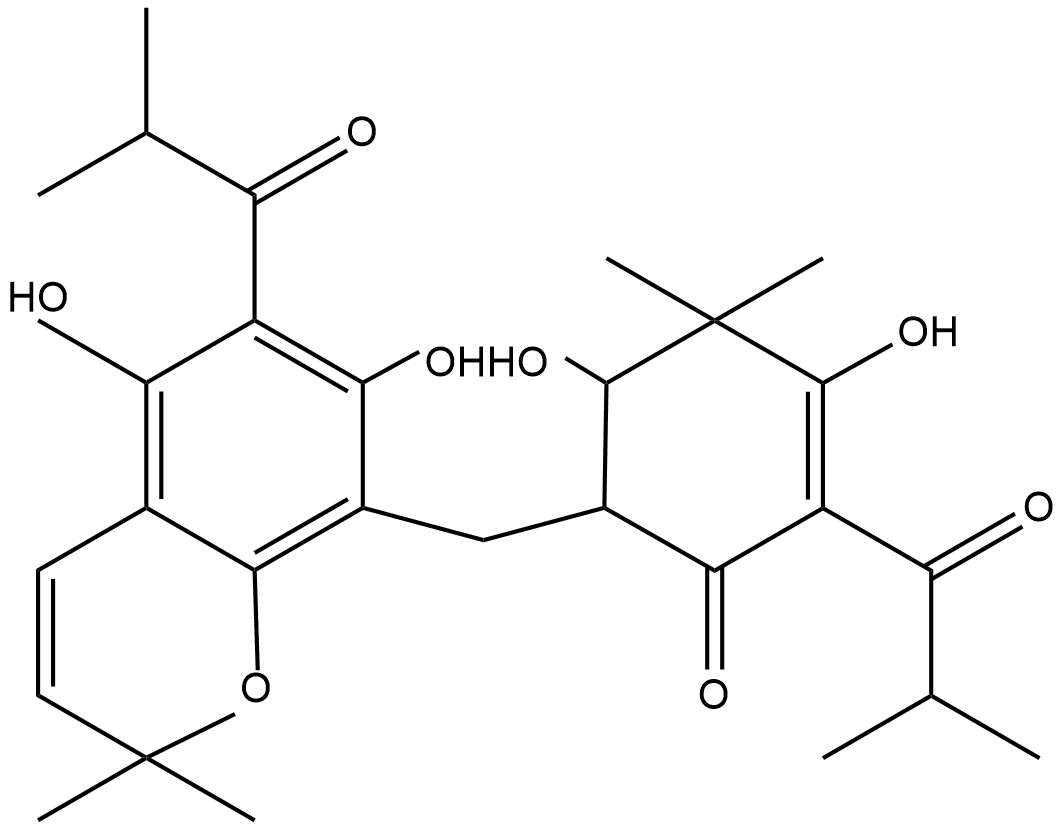  3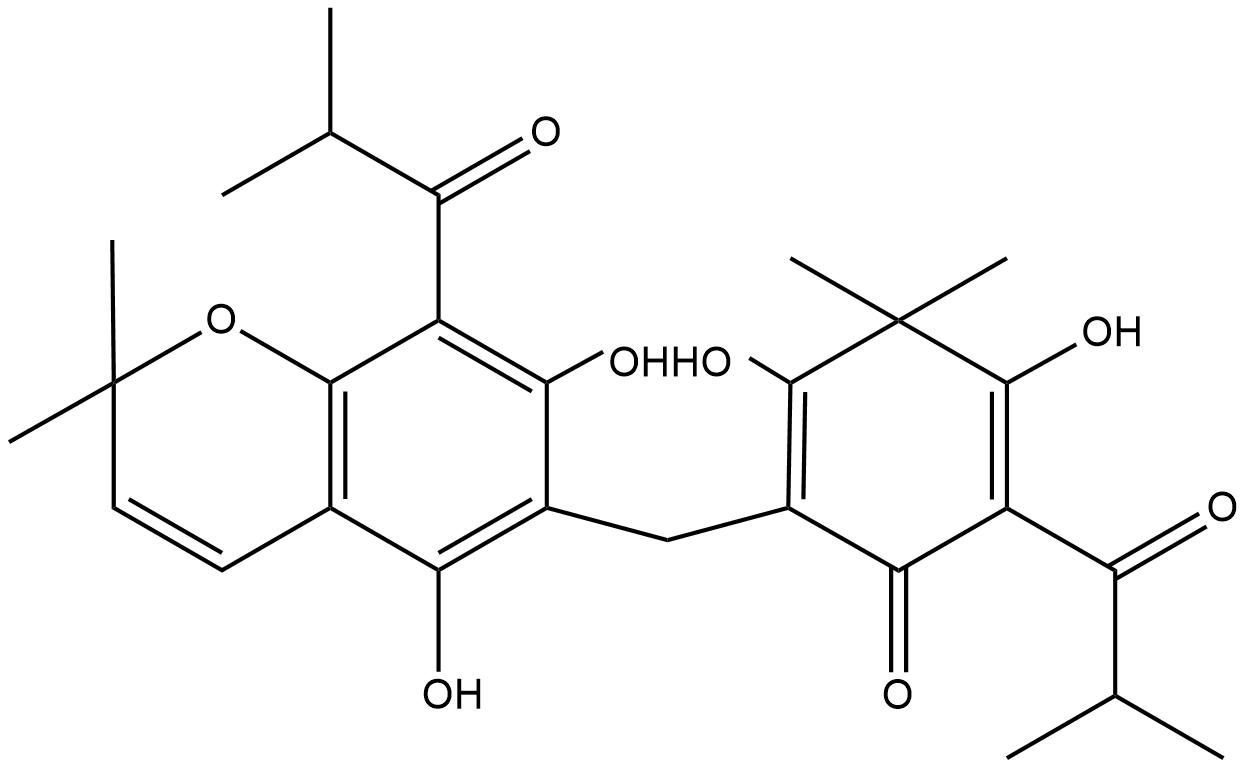 | Inhibition of parasites meadiated by oxidative stress (ROS production) and alteration in mitochondrial potential like hyperpolarization condition. | (Dagnino et al., 2018) |
| 9 | *Euphorbia peplus*  (radium weed) | Peplus aerial parts | Simiarenol | *In vitro*  IC_50_ -20.24, 34.87, 32.05 μg/ml | L. Donovani | 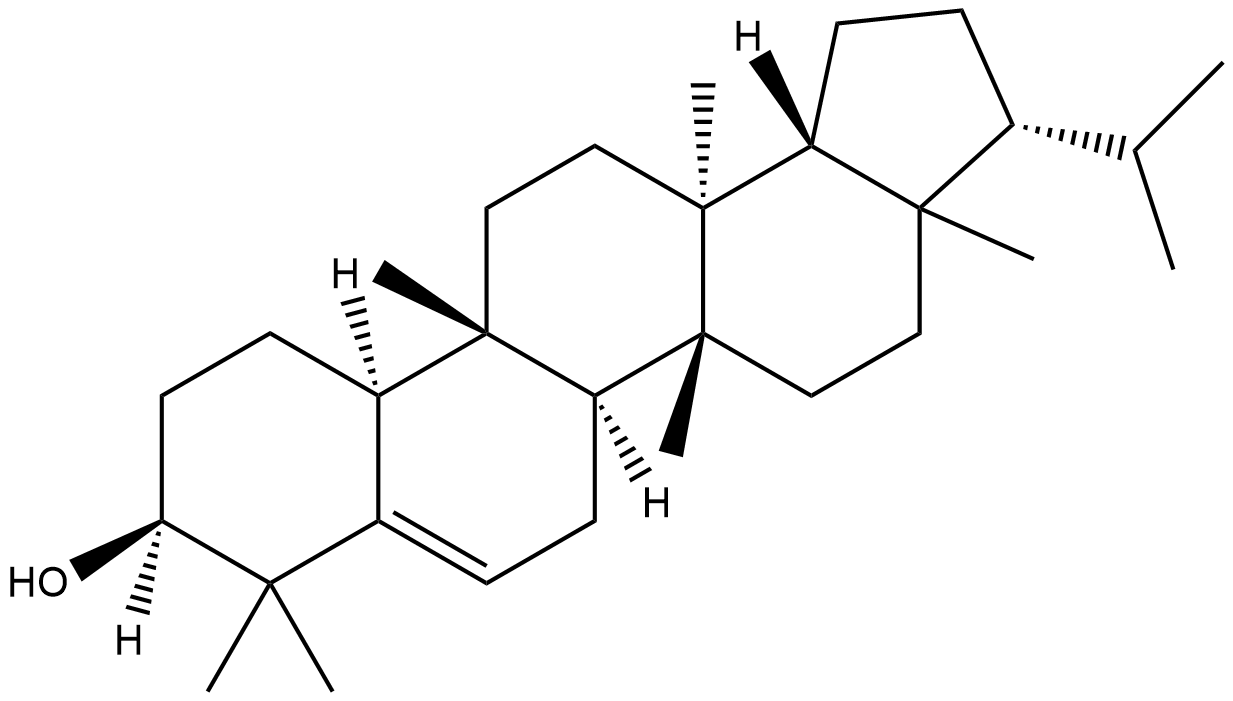 | - | (Moawad et al., 2016) |
| 10 | *Strychnos*  *pseudoquina* | - | Strychnobiflavone | *In vitro*  5.4 and 18.9 μM | *L. amazonensis* | - | Mechanism of action of will be allied with alteration in mitochondrial membrane potential in parasitic cells. |  |
| 11 | Melampodium divaricatum **( butter daisy)**  Casearia sylvestris | Essential oils ( aerial parts of Melampodium divaricatum&  leaves of Casearia sylvestris | E-caryophyllene (22.2%), germacrene D (19.6%) and bicyclogermacrene (12.2%) | *In vitro*  24.2, 29.8 and 49.9 µg/mL respectively | L. amazonensis | 1 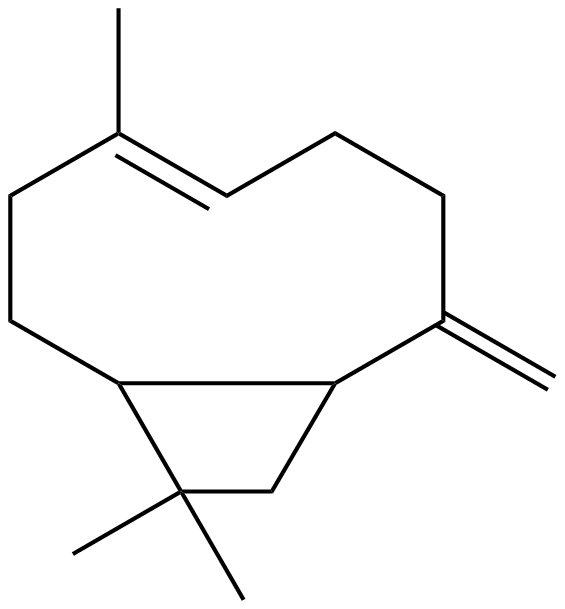  2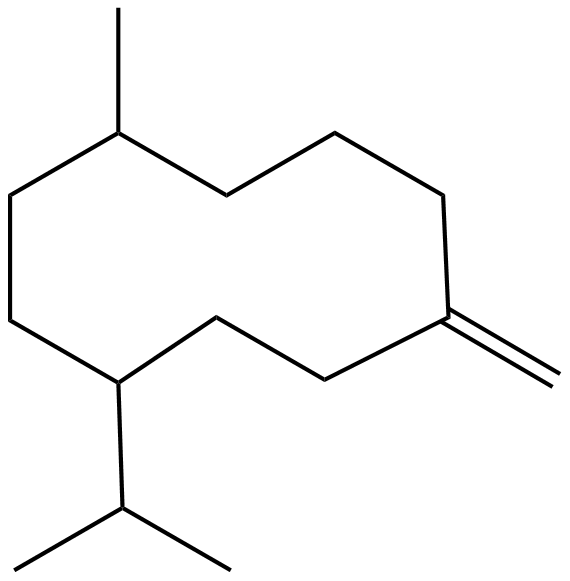  3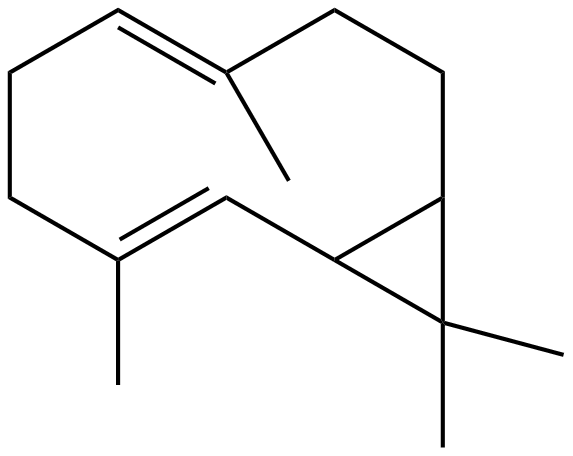 | - | (Moreira et al., 2019) |
| 12 | *Handroanthus* species | - | lapachol | *In vitro & In vivo* (IC_50_ = 79.84 ± 9.10 μM, SI = 42.65) for  *L. amazonensis* and (IC_50_ = 135.79 ± 33.04 μM, SI = 25.08) for *L. infantum*  25mg/kg for *In vivo* model | *L. infantum and L. amazonensis* | 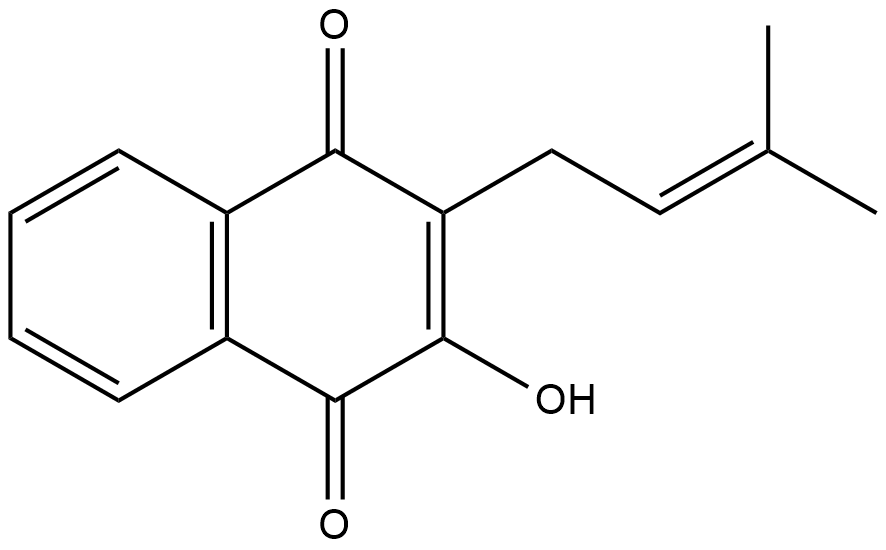 | In *In vivo* model lapachol able to reduce the parasitic load in spleen, liver as well as skin lesions. | (Araújo et al., 2019) |
| 13 | *Ifloga spicata* (I. spicata)  **( alj al anza, alj al ghazal, hasaj)** | whole plant (leaves, flowers, stem, roots) | 3,4-dihydroxybenzoate (compound 1) and benzoate (compound 2) | *In vitro*  LD_50_ values of 10.40 ± 0.09 and 14.11 ± 0.11 µg/mL for compound 1 and compound 2 respectively | *L. tropica*. | 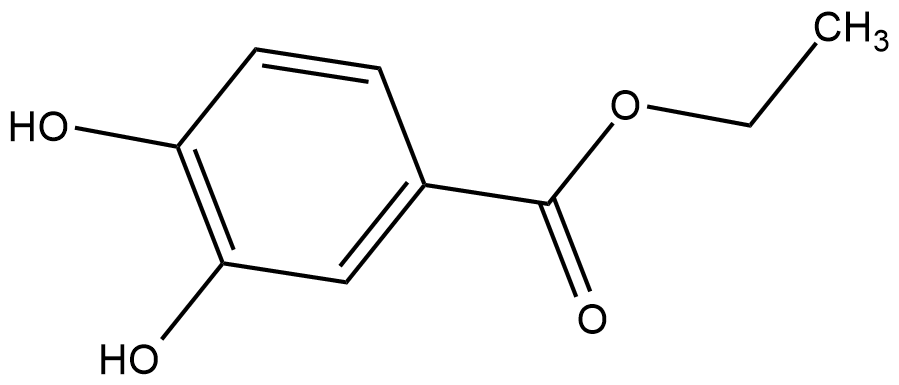  1  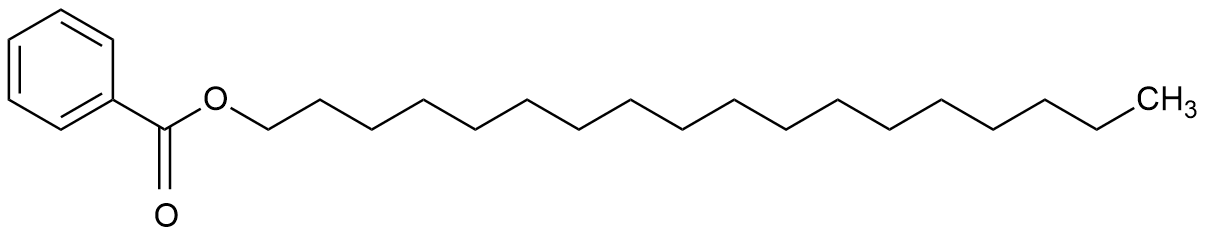  2 | Both the isolated compounds showed great affinity with gp63 (leishmanolysin) receptor of leishmania parasite . Binding of these compounds with their receptors resulted the smooth entry of parasites into the cells and after injection it binds with DNA and causes apoptosis. | (Shah et al., 2019) |
| 14 | *Protium altsonii* (PaEO) and *P. hebetatum* (PhEO) | - | Essential oil | *In vitro* PaEO IC_50_ were 14.8 µg/mL and 7.8 µg/mL and PhEO IC_50_ were 0.46 µg/mL and 30.5 µg/mL | *L. amazonensis* | - | Mitochondrial membrane potential associated with NO production could be a effective mechanism of leishmaniasis . | (Santana et al., 2020) |
| 15 | Artemisia aucheri | Whole plant extract | *-* | *In Vitro & In vivo* IC_50_ 90μg/mL | *L. major* | - | - | (KarimiPourSaryazdi et al., 2020) |
| 16 | *Clerodendrum myricoides* (blue flowered tinderwood) and *salvadora persica* (arak, jhak, pīlu, salvadora indica, toothbrush tree, mustard tree) | Aqueous extract of stem | - | *In vitro*  MIC=625 μg/ml | *L.major* | - | - | (Maina et al., 2020) |
| **17** | Croton blanchetianus Baill. | Ethanolic extract | - | *In vitro* IC_50_ values of 208.6 and 8.8 μg/mL for Leishmania infantum & IC50 values of 73.6 and 3.1 μg/mL for Leishmania amazonensis promastigotes and amastigotes | L. amazonensis and L. infantum | - | Ethanolic extract of Croton blanchetianus targets a significant depolarization of mitochondrial memebrane potential leads to mitochondrial dysfunction. | (Pereira et al., 2020) |
| 18 | Prunus armeniaca (**armenian plum)** | Leaf extract | 1, 2-benzenedicarboxylic acid, diisooctyl ester | *In vitro*  antipromastigotes activity with IC_50_11.48 ± 0.82 μg/ml and antiamastigotes activity with IC50 21.03 ± 0.98 μg/ml | *L. tropica* | 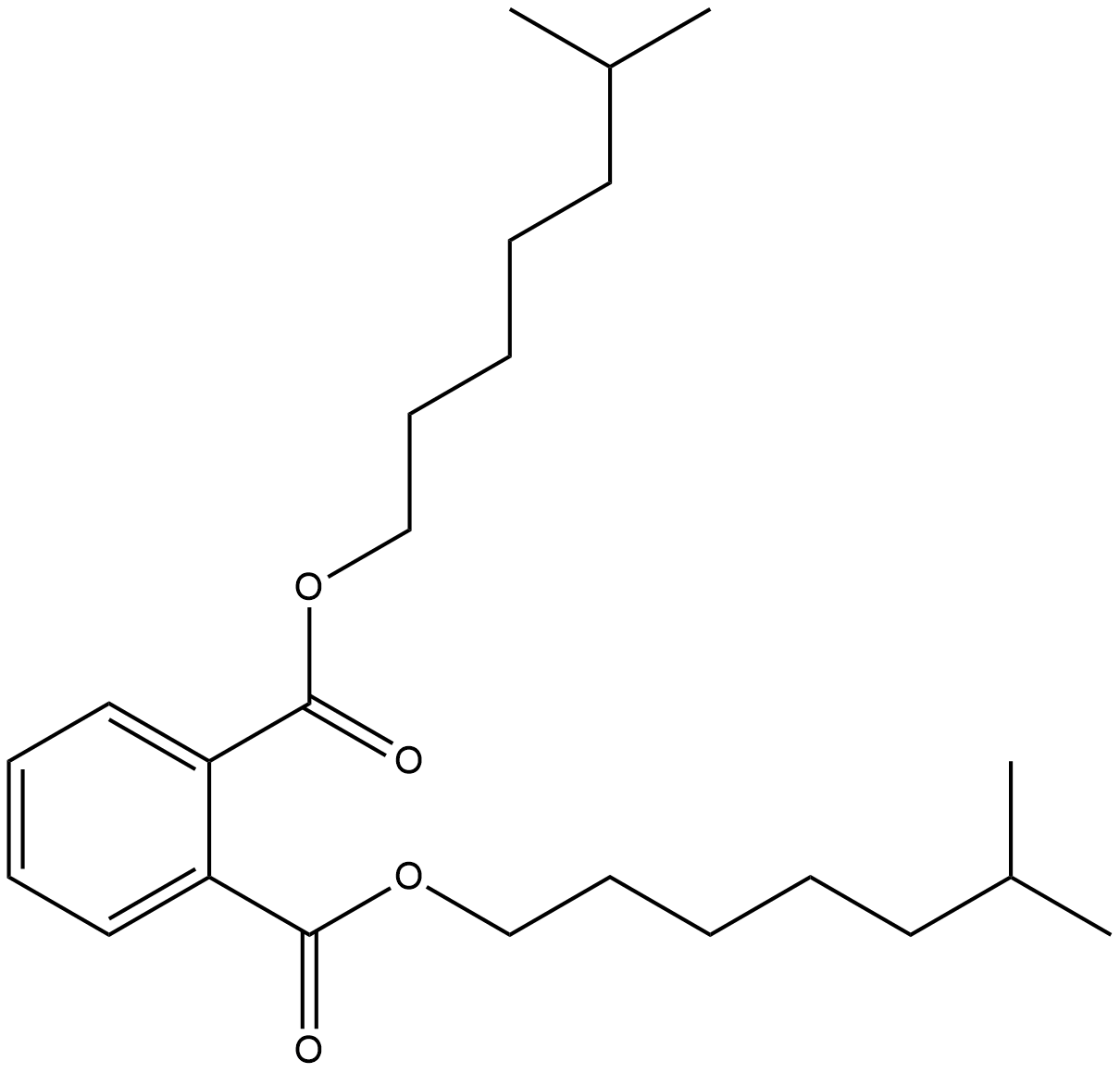 | - | (Shaheen et al., 2020) |
| 19 | Urtica dioica (**common nettle, stinging nettle)** | Aqueous extract | - | *In vivo & In vitro* 3500 and 6000 μg/ml for promastigotes and amastigotes | L. major | - | It proficiently killed the amastigotes form of L. major, additionly remarkable reduction of parasite load, skin lesion size and IL-4 and significant increase of NO and IFN-γ were observed. | (Badirzadeh et al., 2020) |
| 20 | *Piper marginatum* ( cake bush, anesi wiwiri, marigold pepper) | Leaves (ethanolic extract) | 3,4-Methylenedioxypropiophenone | *In vivo* | *L. amazonensis* | 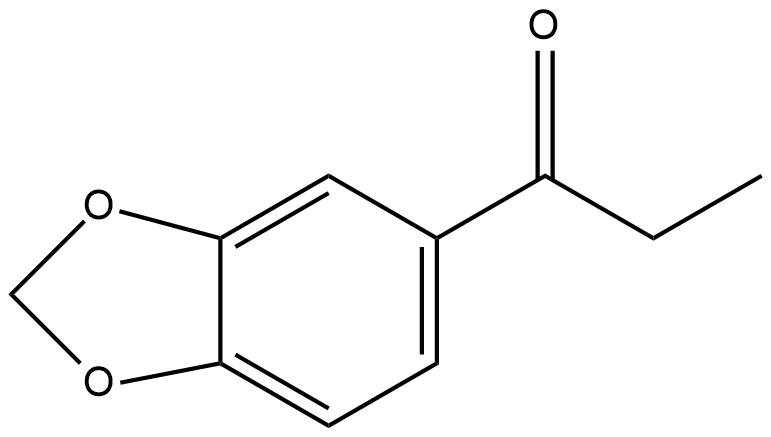 | - | (Macêdo et al., 2020) |
| 21 | *Kelussia odoratissima* ( kelus**celery** and wild celery) | Dried leaves ( butanol fraction) | - | *In vitro* Half (IC_50_) 264.1 and 154.1 µg/mL for promastigotes and amastigotes | *L. major* |  | - | (Mirzaei et al., 2020) |
| 22 | *Tabernaemontana coronaria* ( milkwood) | Dried powder of stem bark | Voacamine | *In vivo*  The IC_50_ value was found to be 14.702 ± 0.101 mM. | *L. donovani* | 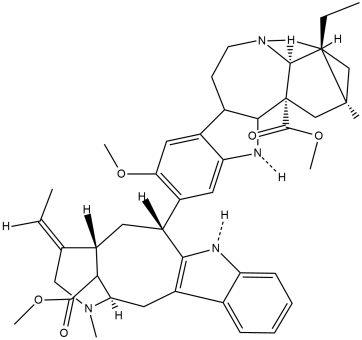 | Voacamine, supress the relaxation potential of LdTop1B (*L. donovani* toposoisomerase IB) as well as steadies the clevable complex. | (Chowdhury et al., 2017) |
| 23 | *Picramnia*  *Gracilis* ( bitterbush) | Powder of dried leaves | 5,3’-hydroxy-7,4’-  Dimethoxyflavanone | *In vitro & In vivo*  EC_50_ 17.0 + 2.8 mg/mL, 53.7 𝜇M for *In vitro* and  2 mg/kg/day for *In vivo* studies | *L. braziliensis.* | 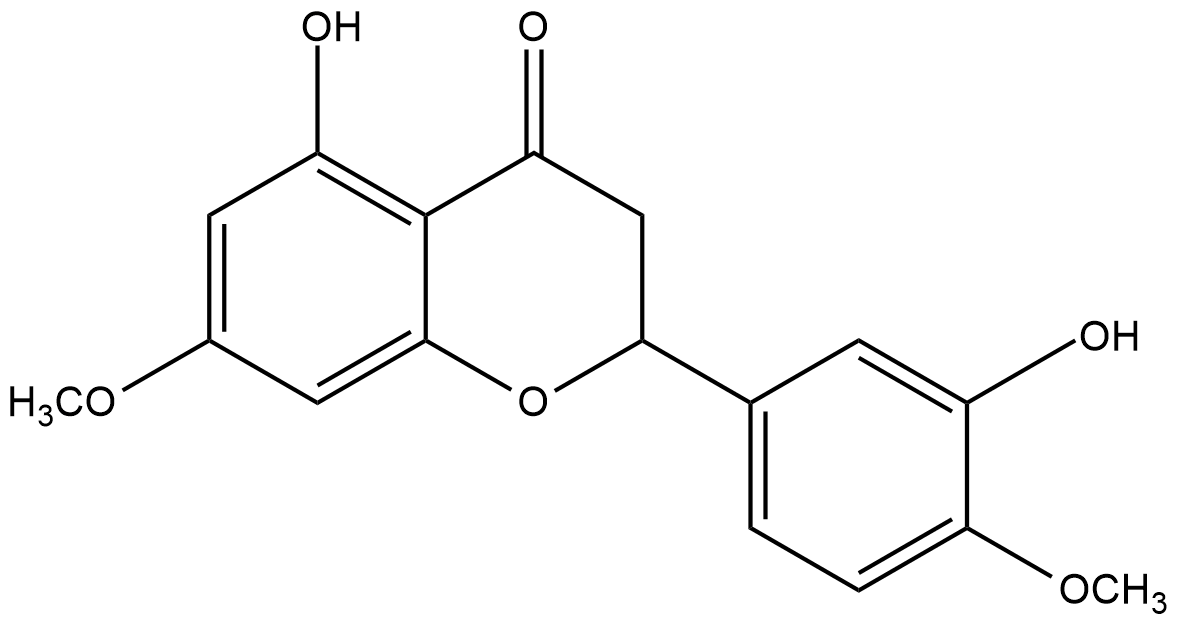 | - | (Robledo et al., 2015) |
| 24 | *Lindera*  *Aggregate*  (spice bush) | Leaves/bark | Boldine | *In vitro* 600 μg/ml | *L. amazonensis* | 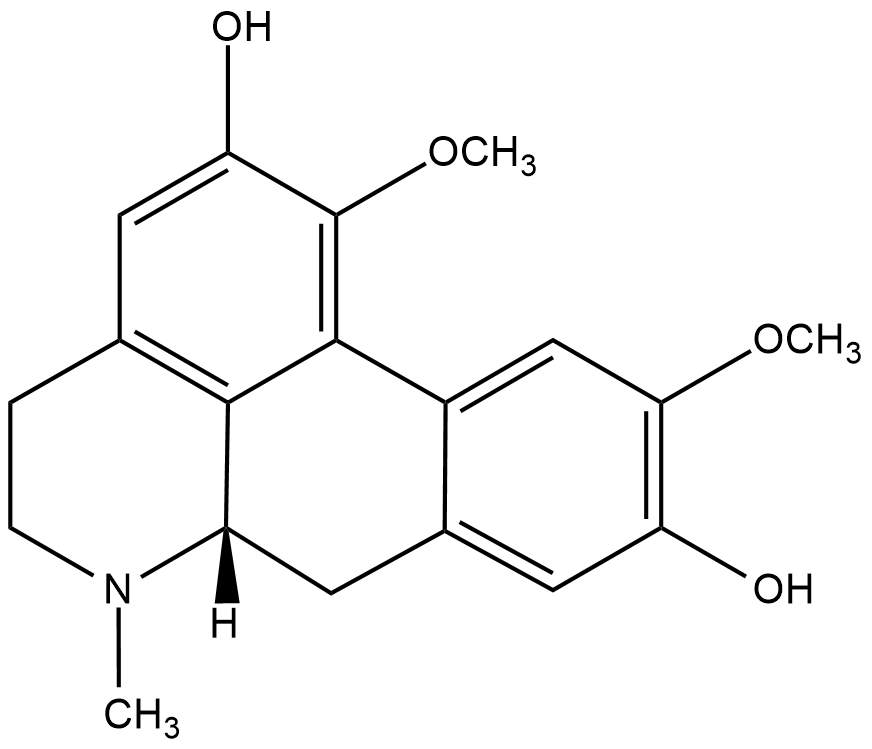 | - | (Salama et al., 2017) |
| 25 | *Hypericum*  *andinum* | Dried and powered materials of aerial parts | Uliginosin  B | *In vitro*  (IC_50_) of 36.1 g/ml | *L. amazonensis* | 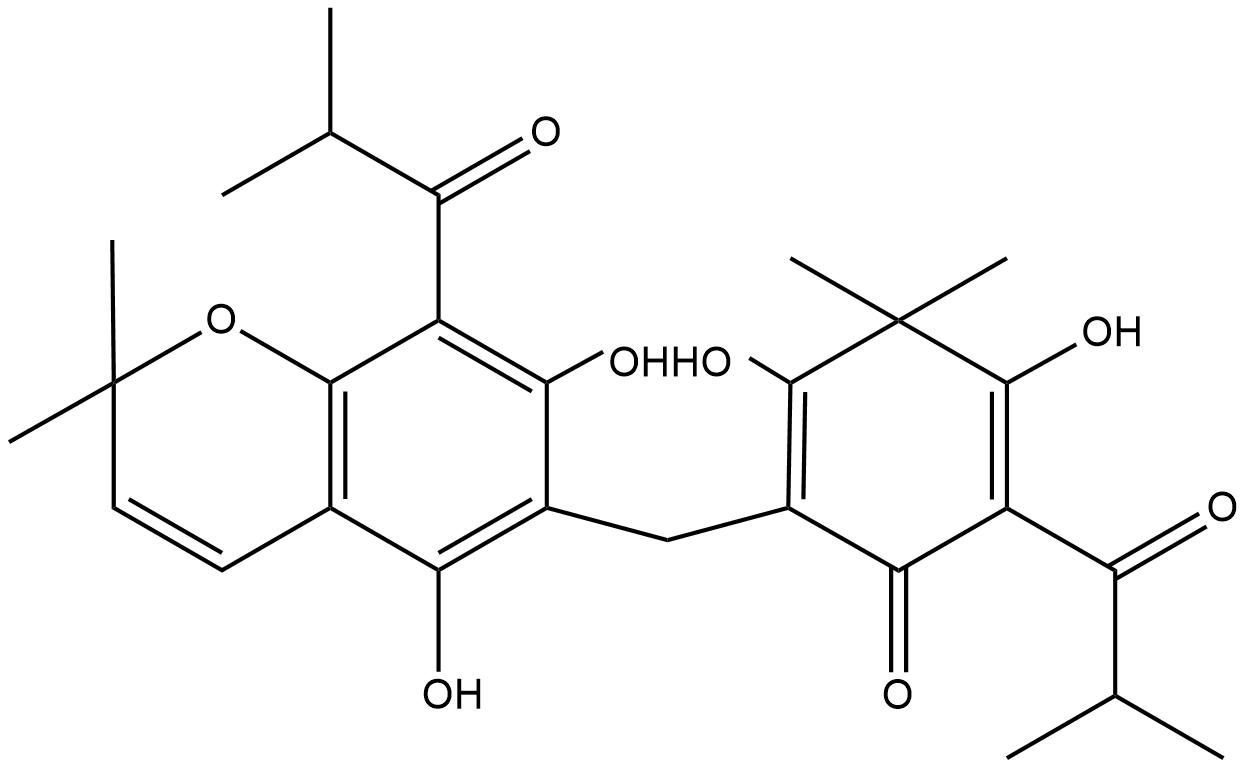 | - | (Dagnino et al., 2015) |
| 26 | *Amphilophium crucigerum* (monkey’s comb) | Aerial parts | Ipolamiide | *In vitro* IC_50_ = 100 µM | *L. amazonensis* | 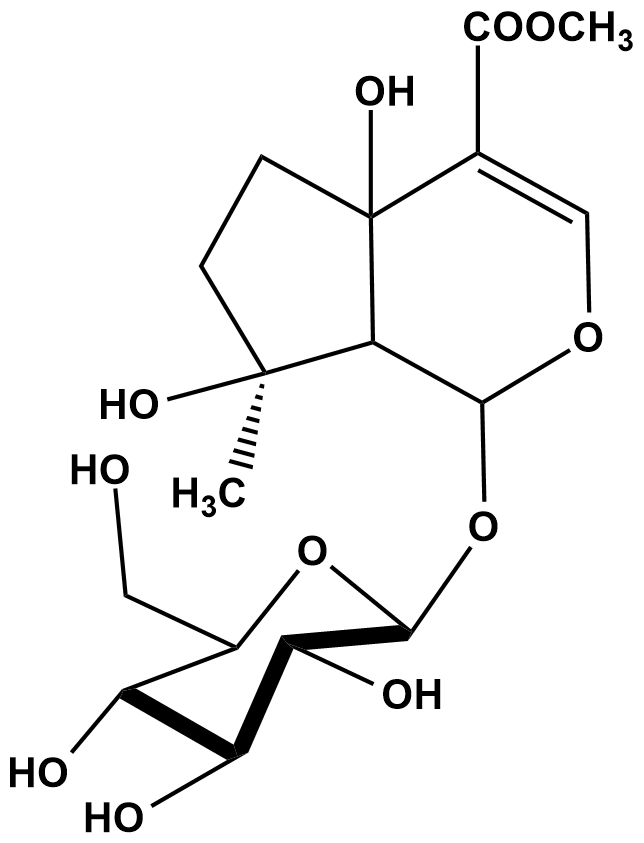 | - | (Vendruscolo et al., 2018) |
| 27 | *Valeriana jatamansi* ( indian valerian or Tagar) | Rhizomes | Valepotriates | *In vitro*  IC_50_ = 2.96 µM | *L. major* | - | - | (Glaser et al., 2015) |
| 28 | *Nymphoides indica* ( **banana plant, robust marshwort** and**water snowflake)** | Leaves | 3-O-methylquercetin-7-O-β-glucoside | *In vitro*  IC_50_ 32 μM | *L. infantum* | 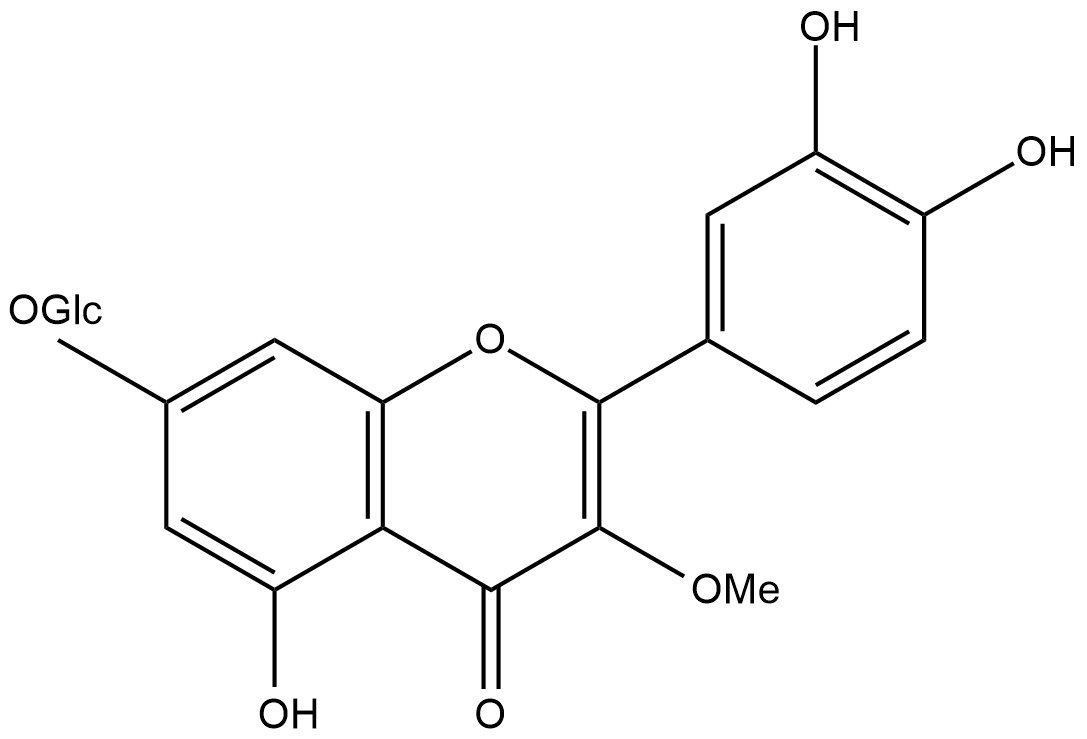 | - | (Amin et al., 2016) |
| 29 | *Vitex grandifolia* ( black plum, Chocolate and berry tree ) | Air dried leaves | Bartioside | *In vitro*  IC_50_ 27.51 µM | *L. donovani* | 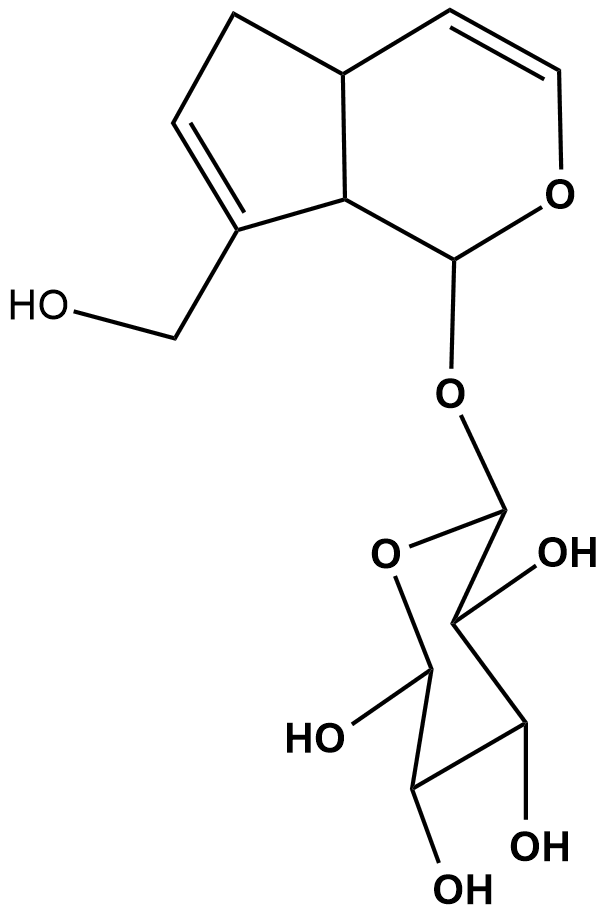 | - | (Bello et al., 2018) |
| 30 | *Scrophularia syriaca* ( **figworts.)** | Aerial parts | 6-O-a-Lrhamnopyranosylcatalpol | *In vitro*  EC_50_ 100 µM | *L.major* | - | - | (Alkhaldi et al., 2020) |
| Section 2  Natural drugs for treating leishmaniasis | | | | | | | | |
| s.no. | Compound (s) | Company/originator | country | Year | Mode of studies | Probable Mechanism involved | Patent & IPC | Ref. |
| 31 | Ethyl 3-(2- chloroacetamido) benzoate, dihydroquercetin and bisabolol | Auclair et al AC Bioscience SA | (Switzerland) | 2019 | *In vitro* and *In vivo* studies | Inhibition of some important parasitic enzymes tryparedoxine peroxidase and tubulin | WO2019043212 & A61K A61P | (Hajaji et al.,2018) |
| 32 | Diterpenoid membranolides | Baker et al University of South Florida | (United States of America) | 2016 | *In vitro* | impedes lipid synthesis | US2016003O388 & A61K | (Baker et al.,2016) |
| 33 | Withaferin-A and Miltefosine | Maurya et al University of Hyderabad | (India) | 2017 | *In vitro* and *In vivo* studies | Inhibits pteridine reductase-1 enzyme, and phosphatidylcholine synthesis, and cytochrome c oxidase. | WO2017046778& A61K A61P | (Maurya and Chandrasekaran., 2016) |
